# The effect of academic supervision, managerial competence, and teacher empowerment on teacher performance: the mediating role of teacher commitment

**DOI:** 10.12688/f1000research.128502.2

**Published:** 2023-09-19

**Authors:** Imron Muttaqin, Nani Tursina, Ajat Sudrajat, Uyung Yuliza, Novianto Novianto, Fajar Fahri Ramadhan, Muhammad Edi Kurnanto

**Affiliations:** 1 Fakultas Tarbiyah dan Ilmu Keguruan, Pontianak State Institute for Islamic Studies, Pontianak, Kalimantan Barat, 78122, Indonesia; 2 Postgraduate Program, Pontianak State Institute for Islamic Studies, Pontianak, Kalimantan Barat, 78122, Indonesia; 3 Fakultas Tarbiyah dan Ilmu Keguruan, Instisut Agama Islam Negeri Pontianak, Pontianak, Kalimantan Barat, 78122, Indonesia; 4 Fakultas Ushuluddin Adab dan Dakwah, IAIN Pontianak, Pontianak, Kalimantan Barat, 78122, Indonesia

**Keywords:** Academic supervision, principal managerial competence, teacher empowerment, teacher commitment, teacher performance

## Abstract

**Background**: Teacher commitment and performance are important factors contributing to student achievement and the quality of education. Therefore, it is critical to draw attention to this issue by identifying factors that influence these two variables.

**Methods**: A survey, consisted of 34 closed-ended questions, was used. The total sample comprised 2,203 teachers, including 832 men and 1,371 women. Data analysis was performed using partial least squares (PLS) structural equation modeling (SEM). Model measurement employed indicator loading, convergent and discriminant validity, Cronbach's testing, composite reliability and average variance extracted (AVE) for internal consistency. Structural model measurement employed coefficient determination (R2), effect size (F2), variant inflation factor (VIF), predictive relevance (Q2) and path analysis measures.

**Results**: The results of the study reveal that academic supervision has a significant positive effect on commitment (β; 0.085, t-statistics; 3.792 and p-value 0.000), academic supervision also affects teacher performance (β; 0.096, t-statistics; 4.416 and p-value 0.000), managerial competence affects teacher commitment (β; 0.195, t-statistics; 8.046 and p-value 0.000), managerial competence also affects teacher performance (β; 0.149, t-statistics; 6.561 and p-value 0.000), organizational commitment affects teacher performance (β; 0.163, t-statistics; 7.263 and p-value 0.000), teacher empowerment affects teacher commitment (β; 0.489, t-statistics; 22.601 and p-value 0.000), and teacher empowerment affects teacher performance (β; 0.489, t-statistics; 22.601 and p-value 0.000). Academic supervision affects teacher commitment mediating by teacher performance (β; 0.014, t-statistics; 3.178 and p-value of 0.001). Managerial competence affects teacher commitment with teacher performance mediation (β; 0.032, t-statistics; 5.588 and p-value 0.000), and teacher empowerment affects teacher commitment with teacher performance mediation (β; 0.080, t-statistics; 7.050 and p-value 0.000).

**Conclusion**: Academic supervision, managerial competence, and teacher empowerment significantly positively affect teacher performance directly and through teacher performance as mediating variable. Teacher empowerment is the most significant factor influencing teacher performance and commitment.

## Introduction

The World Bank report inform that teacher competence and performance in Indonesia remain suboptimal, thereby indicating the need for competency strengthening, continuous professional development, and integrated performance and performance appraisal ( Yarrow et al., 2020). Teacher performance is a crucial issue warranting special attention. It is intimately connected to a teacher’s commitment, as these two aspects are inextricably linked ( Mart, 2013; Somers & Birnbaum, 2000). Commitment refers to a teacher’s dedication to their duties and responsibilities. Teachers demonstrating high commitment are likely to exhibit elevated performance levels. This strong attachment to their responsibilities can significantly impact their performance. Therefore, if there is indeed a close correlation between teacher performance and commitment, improvement in one variable should yield an increase in the other. Although several previous studies have substantiated the impact of commitment on performance, it remains relatively rare for studies to treat commitment as a mediating variable between other factors influencing teacher performance. Consequently, research is needed to ascertain the effect of commitment, whether directly or indirectly, in relation to other variables.

The principal plays a pivotal role in assessing and enhancing teachers’ performance and commitment. Studies conducted on 65 teachers in junior high schools and vocational schools reveal that the leadership model significantly influences teachers’ performance ( Kosim, 2017). Similarly, a separate study conducted in Prabumulih with 99 junior high school teachers affirmed the principal’s influence on performance ( Jaliah et al., 2020). For primary school teachers, evidence also exists of a positive influence ( Zuldesiah et al., 2021). Notably, all three studies were conducted in schools under the supervision of the Ministry of Education and Culture. Principal leadership is associated with managerial skills and involves motivating and empowering teachers, along with providing teachers academic supervision and support.

Numerous prior studies have elucidated the impact of various variables on teacher performance. For instance, study demonstrated the effect of professional training on teacher performance ( Guthrie, 1982), Subsequently, identified key dimensions of teacher performance, namely occupational morality, job dedication, assistance and cooperation, teaching effectiveness, teacher-student interaction, and teaching value ( Cai & Lin, 2006). Further research has established connections between performance pay, teacher certification, motivation, school climate, and teacher performance ( Hill & Jones, 2018). In Abu Dhabi, the influence of professional development training on teacher performance has been investigated ( Afi, 2019). Additionally, a study involving 213 teachers from 26 Indonesian provinces suggested that teacher performance could serve as an intermediary variable influencing student learning outcomes ( Amtu et al., 2020). Other studies have explored the relationships between supervision ( Surayya & Sumirah, 2021), professional allowances, teacher professionalism ( Farezhena & Wijayaningsih, 2022), and management and motivation on teacher professionalism ( Amyanih & Sussanto, 2022).

A case study conducted in Pakistan indicated that distributed leadership could foster teacher commitment ( Berjaoui & Karami-Akkary, 2020), Concurrently, principal competence was found to impact job satisfaction and teacher job commitment ( Rachmawati & Suyatno, 2021). The significance of supervision competence, which is a key attribute for principals, has also been underlined. Related research has shown that supervision impacts teacher commitment ( Kusumawati, 2020). In addition to influencing commitment, supervision also impacts teacher performance. A systematic review article spanning the years 2015-2021 demonstrated the direct effect of principal supervision on teacher performance ( Imamah & Churrahman, 2022).

*RQ 1*: Does academic supervision have a significant positive effect on teacher commitment?

*RQ 2*: Does academic supervision have a significant positive effect on teacher performance?

The principal plays a pivotal role in enhancing the quality, competence, and performance of teachers. The ability of the principal to manage the attainment of the vision and mission, as well as the utilization of all school resources, is integral to the progress of the school. The commitment and performance of teachers are substantially influenced by the principal. Al-Mahdy found that the principal’s instructional leadership significantly impacts teacher commitment ( Al-Mahdy et al., 2018). Teacher commitment refers to the teacher’s dedication to his or her duties and responsibilities. Furthermore, commitment is an intervening variable in the influence of school-based management on teacher performance ( Haitami et al., 2022). The influence of the principal on teachers is indeed profound ( Albuni, 2022; Chomariah et al., 2022; Nizar, 2021; Nurhasyim et al., 2021; Timang et al., 2021), The principal’s capability to manage schools has a bearing on teacher performance ( Fatkurinah, 2020; Suhardi, 2019). In the final analysis, teachers who demonstrate high commitment are likely to exhibit high performance, as indicated in a study involving 93 teachers, which revealed the impact of teacher compensation and commitment on teacher performance ( Setiawan et al., 2020). Based on the findings of these studies, the following research questions have been formulated:

*RQ 3*: Does the managerial competence of the school principal influence teacher commitment?

*RQ 4*: Does the managerial competence of the school principal influence teacher performance?

*RQ 5*: Does teacher commitment have a significant positive effect on teacher performance?

The important role of the principal is not limited to achieving the vision and mission using existing facilities and infrastructure, but also extends to developing the school’s human resources. One form of such development is the empowerment of teachers. Empowerment involves the provision of power, flexibility, training, and access, enabling teachers to develop in accordance with their competencies ( Ahmad et al., 2014), Another study involving 356 teachers at Payam E-noor University demonstrated a positive influence of teacher empowerment on teacher commitment ( Aliakbari & Amoli, 2016; Lyons et al., 2013).

*RQ 6*: Does teacher empowerment have a significant positive effect on teacher commitment?

*RQ 7*: Does teacher empowerment have a significant positive effect on teacher performance?

Academic supervision is purposed to support teachers in the effective execution of their duties and responsibilities. It is hypothesized that such supervision influences teacher performance indirectly, through mediating the teacher’s commitment. A study conducted on 152 teachers in Ghana revealed a correlation between clinical supervision, a particular type of academic supervision, and teacher commitment ( Owusu-Addo et al., 2022), A wealth of research has established the influence of commitment on teacher performance ( Supriyanto, 2022). Consequently, an enquiry is warranted regarding whether teacher commitment also serves as a mediating variable in the relationship between academic supervision and teacher performance.

*RQ 8*: Does academic supervision have a significant positive effect on teacher performance through the mediation of teacher commitment?

The influence of principal communication management on teacher performance is mediated by both psychological empowerment and teacher affective commitment ( Yao et al., 2020). Communication skills constitute a crucial element in managerial competence. Principals who can effectively articulate their vision and mission, provide leadership, clarify job descriptions, and motivate teachers are naturally likely to enhance teacher performance. A strong commitment to duties and responsibilities can enhance teachers’ dedication, leading to superior teaching outcomes. Consequently, it is reasonable to hypothesize that teacher commitment could serve as a mediating variable of the principal’s managerial competence.

*RQ 9*: Does managerial competence have a significant positive effect on teacher performance through the mediation of teacher commitment?

Teacher commitment serves as a mediating variable in the influence of teacher empowerment on teacher performance ( Limon, 2022). The instruments utilized in this study were adapted from Meyer’s indicators, including affective commitment, continuance commitment, and normative commitment. Furthermore, various other studies have demonstrated that teacher enthusiasm and empowerment are also mediated by teacher commitment, thus exerting a positive influence on teacher organizational behavior ( Cheasakul & Varma, 2015). In the context of the business world, research has also been conducted, the findings of which revealed that commitment can serve as a mediating variable of empowerment, even in the industrial sector ( Akhtar et al., 2020).

*RQ 10*: Does teacher empowerment have a significant positive effect on teacher performance through the mediation of teacher commitment?

Based on the literature outlined above, it remains few research focused on teacher commitment as a mediating variable for teacher performance. Therefore, the research question for this study has been formulated as follows: Do academic supervision, managerial competence, and teacher empowerment exert a direct influence on teacher performance and an indirect influence via the mediation of teacher commitment?

Teacher commitment and performance are the keys to successful learning because they directly impact students’ commitment ( Abu Nasra & Arar, 2020; Sun, 2015) and mediate the quality of work and performance. Teacher commitment is also influenced by teacher performance ( Singh, 2022), but there is still little research that discusses the effect of performance on teacher commitment. Meanwhile, teacher empowerment is influenced by several factors: teacher commitment is indispensable to a school ( Shirrell, 2016), and a school can also contribute to the improvement of commitment with reasonable management efforts ( Karakuş & Aslan, 2009). Teacher empowerment is also influenced by the principal’s managerial competence and supervision.

Research in this area is important because a) few studies link managerial competence, supervision, and empowerment to teacher commitment and b) improving professionalism and teacher performance in Indonesia is pivotal, necessitating an assessment of the relationship between variables that affect professionalism and teacher performance and commitment. Indonesia also needs teachers’ commitment and performance to improve the quality of education. Given the rationality of the importance of factors affecting teacher performance and commitment, it was interesting for our research team to test these factors. The present study focused on teachers based in the West Kalimantan and East Java Provinces. Teachers in West Kalimantan Province were chosen because of the province’s Pontianak State Institute for Islamic Studies system, which requires teachers to synergize and participate in facilitating teachers’ development. The province of East Java was chosen because it has the highest concentration of teachers in Indonesia.

## Literature review and theoretical framework

### Academic supervision

Supervision is a service to teachers that aims to improve the learning, methods, and curriculum used to achieve goals. In general, educational supervision involves coaching toward improving educational services through guidance and enhancing and improving the quality of teaching. Such education provides guidance to improve educational services in general and the quality of teaching and learning ( Ametembun, 1991). A supervisor must have personality, managerial, academic, educational evaluation, research, and social competencies as stated in the Regulation of the Minister of Education and Culture Number 12 of 2007 concerning School/Madrasah Supervisory Standards ( Nasional, 2007). Supervision has several functions: research, assessment, and improvement. Academic supervision significantly affects teacher performance ( Prasetyono et al., 2018) and teacher empowerment ( Davis & Wilson, 2000).

Principal supervision affects teacher performance ( Magdalena et al., 2020). They also state that supervision from the principal is very important in improving teachers’ performance and commitment because teachers with such supervision feel guided by their superiors. Other studies have also shown similar results; principal clinical supervision, work motivation, work climate, and teacher professional benefits affect teacher job satisfaction ( Febrina Subagia et al., 2019). Teacher performance is influenced by supervision ( Veloo & Zolkepli, 2011).

**Managerial competence**

The managerial competence of the principal is a form of competency that includes the ability to plan; carry out organizational development; lead and utilize school resources and manage changes, development, and learning in schools such that they are more effective and efficient in achieving the school’s vision and mission. Previous studies have consistently shown that principal leadership affects teachers’ and students’ quality of learning and motivation ( Muijs & Harris, 2003). In addition, a principal needs to strive for teacher participation based on his knowledge ( Richardson, 2003). Previous studies have revealed that the leadership model affecting teacher empowerment is participatory because it positively impacts teachers’ impact, autonomy, self-efficacy, and job satisfaction ( Martino, 2003) and is a mediating variable for teachers’ commitment to schools ( Bogler & Somech, 2004). A principal’s leadership also affects teacher performance ( Satriadi, 2016). Managerial competence is part of principal leadership ( Rinehart et al., 1998). Previous research discusses the effects of teacher managerial competence and teacher commitment ( Ben Sedrine et al., 2021), but few studies discuss such effects in reference to the West Kalimantan and East Java Provinces.

Managerial competence for principal very important, competence is an individual characteristic of effective or superior reference criteria applying to specific work situations ( Spencer and Spencer, 2008), or characteristics that appear on the surface include skills and knowledge, while those that are not visible include motivations, traits, self-image, and social roles. These invisible characteristics largely determine a principal’s success in leading and influencing his actions and thoughts. A principal must exhibit managerial communication skills, teamwork, proactiveness, vision, self-management, outcome orientation, strategies, perseverance, decision-making skills, risk-taking, and creativity ( Bhardwaj & Punia, 2013). Such abilities strongly support the tasks and functions of leadership in an organization.

Other studies report that managerial competence comprises the ability to analyze, make decisions, apply knowledge, adapt, perform, lead, and communicate (Khoshouei et al., 2013). A principal as an excellent manager has competence in the following areas: 1) impact and influence, 2) achievement orientation, 3) teamwork and cooperation, 4) analytical thinking, 5) initiative, 6) developing others, 7) self-confidence, 8) directive/assertiveness, 9) information searching, 10) team leadership, and 11) conceptual thinking ( Spencer and Spencer, 2013). Previous studies have shown that managerial competence is related to performance ( Victor, 2017), and the managerial competence of principals and compensation affect teacher performance ( Suhardi, 2019). Research by Lourena Fitri May *et al.* (2020) also proves the effect of managerial competence on teacher performance. Other factors affecting teacher performance include the organizational climate ( Jannah et al., 2019) and leadership styles ( Verawati Wote & Patalatu, 2019). However, these two variables are not considered in this study.

**Teacher empowerment**

Teacher empowerment is an effective means to achieve the vision and mission of education and academic and nonacademic achievement in schools. Teacher empowerment is essentially an effort to support teachers in upholding their duties and responsibilities independently. The significant power of teachers affects many aspects of education, such as motivation, performance, student achievement, job satisfaction, and school achievement. Teacher empowerment can be divided into psychological and structural empowerment. Psychological empowerment affords one a feeling of being able to carry out one’s work well, which has dimensions of meaning, competence, self-determination, and impact ( Spreitzer, 1995). Teacher empowerment, according to Short and Rinehart (1992) includes six dimensions: involvement in decision-making, impact, status, autonomy, professional development opportunities, and self-efficacy. Teacher empowerment affects teacher professionalism and the school itself ( Moore & Esselman, 1992; Patrick et al., 2003). In addition, teacher empowerment is very important for the government ( Reitzug, 1994) because it can improve the quality of national education and positively influence teachers’ professional and organizational commitment ( Bogler & Somech, 2004), which in turn can affect the quality of education. Teacher empowerment is carried out by providing teachers with the power to conduct their duties and responsibilities and continuously improve their competencies to influence their organizational behavior ( Tindowen, 2019), job satisfaction ( Nursyfa & Asmawi, 2018) and mindset development ( Seaton, 2018).

Teacher empowerment is the provision of power to teachers to have power, autonomy, choice, responsibility, and status regarding their duties as teachers. To determine the empowerment of teachers in schools, researchers have uncovered the dimensions of teacher empowerment. Melenyzer (1990) states that there are four dimensions of empowerment: organizational culture, social practice with the strengthening of social norms, and the use of transformational leadership. Wunder (1997) added the professional community as a dimension of teacher empowerment.

These dimensions include instruments specifically used to measure teacher empowerment, including the School Participant Empowerment Scale (SPES) proposed by Short and Rinehart (1992). In 1998, confirmatory factor analysis was conducted to retest the SPES indicators on 4,091 teachers. The study results showed the need for revisions to the six subscales used by Short and Rinehart ( Klecker & Loadman, 1998), and Klecker and Loadman (1998) then made his instrument to measure teacher empowerment.

Previous research findings reveal a relationship between teacher empowerment and joint decision-making ( Bernd, 1992). Delegating decision-making power to teachers has also been shown to increase teachers’ organizational commitment ( Mohd Ali & Yangaiya, 2015). Additionally, the involvement of teachers as a decision-making team ( Dee et al., 2006) makes them part of the leadership team ( Srivastava & Dhar, 2016) because the involvement of teachers as members of this team affects their job satisfaction where teachers’ job satisfaction ultimately affects their empowerment. Teacher empowerment can also be achieved by forming a teacher work team ( Park, 2004).

Teacher empowerment can be achieved through many activities, such as cognitive training ( Cochran & DeChesere, 1995), decision-making involvement, and self-efficacy improvement ( Crum, 1996). The continuous professional development of teachers needs to shift from a mechanistic technical approach to a model of social change that is integrated with the community, schools, and teachers ( Patrick et al., 2003), and also more specifically, with the culture of local communities. Many research results reveal that in Indonesia, teacher empowerment activities include writing scientific papers ( Chairunnisa, 2016), utilization of information and communication technology ( Chodzirin, 2016), teacher certification ( Permana, 2017), and sustainable professional development ( Pratama, 2018; Rohmah, 2018).

Self-efficacy refers to a teacher’s confidence in his ability to manage and decide what needs to be done when facing problems related to his tasks. Teachers who do not have confidence in their abilities will see an effect on their performance and job ( Skaalvik & Skaalvik, 2014). Teachers’ self-efficacy in some countries can differ according to their cultural orientation ( Vieluf & Göbel, 2019), self-efficacy measurements must be modified to a specific country’s culture. Previous research has shown that teacher self-efficacy affects teacher job satisfaction ( Klassen et al., 2018), and teacher job satisfaction affects teacher performance ( Damrus, 2018; Julianingsih & Paramartha, 2018). Another study showed that job satisfaction affects the performance of junior high school teachers in Sekayu ( Widayati et al., 2020).

**Teacher commitment**

Commitment is an important trait for any teacher; teaching with full commitment can be judged by the passion and enthusiasm of the teacher when teaching. Commitment is here defined as exhibiting dedication to the goals and values of an organization relating to the role of the teacher in achieving learning objectives. Teachers with high commitment are determined and responsible in their teaching. This attitude is needed to improve student achievement. The term commitment comes from social exchange theory, which underlies the emergence of social relationship theory due to the development of needs in organizations ( Cropanzano & Mitchell, 2005). The theory states that social exchange involves continuous interaction that creates obligations ( Emerson, 1976).

Furthermore, social exchange theory emphasizes an individual’s contribution to an organization and participation ( Angle & Perry, 1981). The theory is ultimately the foundation of organizational commitment theory. In this case, a teacher’s commitment and interaction with the school and its students are at the core. Steers and Spencer (1977) explain that commitment refers to an individual’s positive orientation toward his organization; a teacher behaves positively toward the tasks assigned to him at school. Steers and Spencer stated that there are three indicators of commitment: the desire to remain in the organization, to strive according to the organization’s wishes, and to accept the organization’s values and goals.

Some studies show that teacher commitment is influenced by the leadership of the principal ( Yu et al., 2002), leadership behavior ( Huang, 2011), authoritarian leadership ( Parlar et al., 2022), transformational leadership ( Yahaya & Ebrahim, 2016), and distributed leadership ( Liu, 2020). Some of the studies above highlight the principal’s leadership role, which requires good managerial abilities to ensure the quality of education. Therefore, it is interesting to examine whether managerial competence affects teacher commitment. In addition, teacher involvement also increases commitment ( Jyoti et al., 2021). As an important dimension of empowerment, we explore whether teacher empowerment can affect teacher performance through teacher commitment.

**Teacher performance**

Performance refers to a person’s achievement in carrying out their duties and obligations based on authority and responsibility. Kane (1986) defines performance as performing one’s position in a certain period, while Otley (1999) argues that performance is only intended for the business and public sectors because it relates to effectiveness, efficiency, and economy in work. Teacher performance can be articulated as a result of a teacher’s work in carrying out his teaching duties, from planning and implementation to evaluation/assessment.

**Figure 1.**
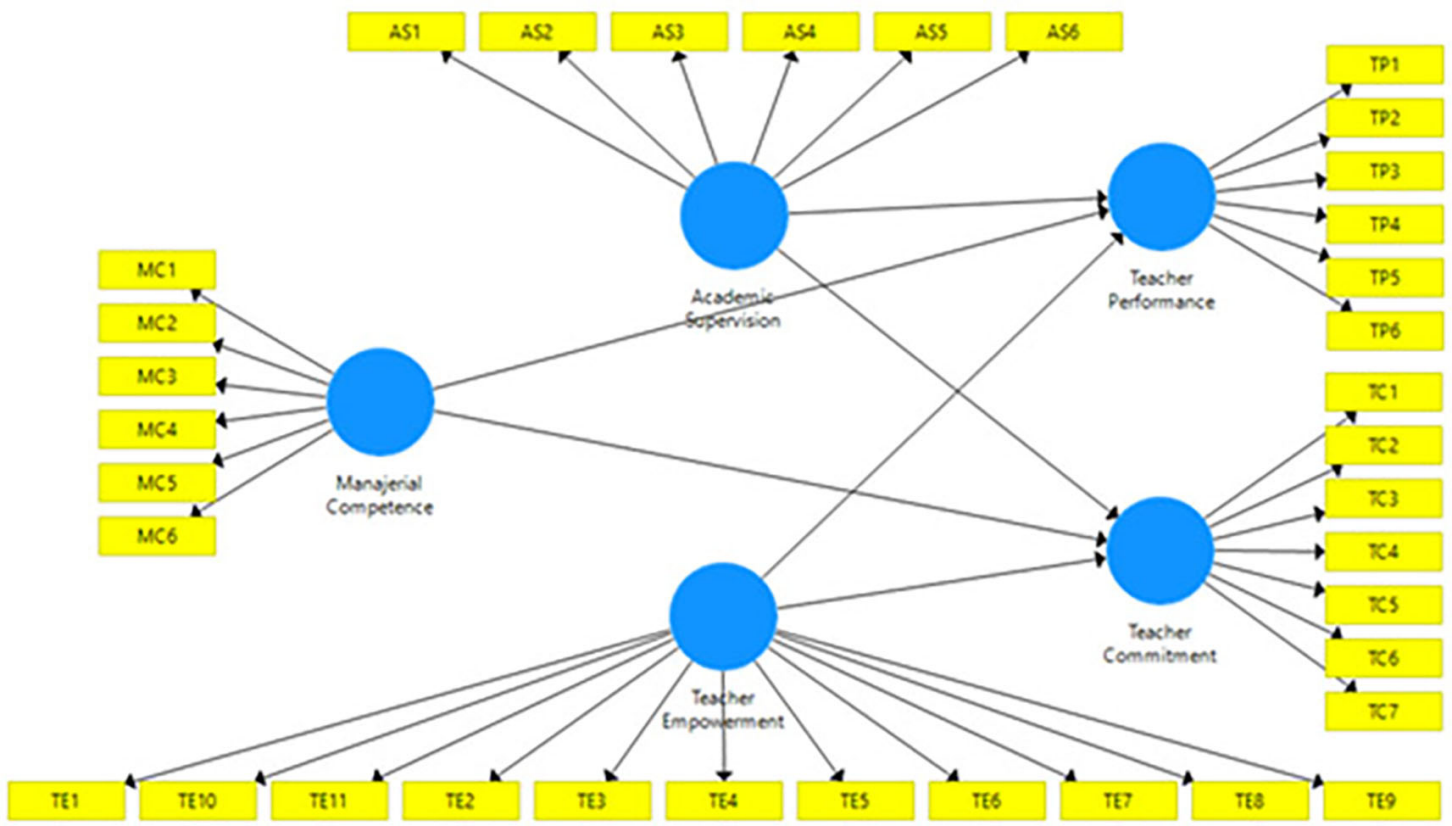
Proposed model.

Teacher performance is influenced by several factors, including the leadership of the principal ( Abu Nasra & Arar, 2020), which should be supported by several competencies to be done well. One of these competencies is managerial competence, which is needed for a school to be able to organize and achieve its vision and mission. Teacher job satisfaction ( Wolomasi et al., 2019), commitment, and performance are also influenced by teacher job satisfaction ( Fei & Han, 2019). Teacher performance is related to the tasks assigned to teachers ( Sultana, 2020); tasks that demand high performance are also often responded to with high performance. The duties and responsibilities of a teacher require supervision and meeting targets that have been set. Therefore, academic supervision is needed to help teachers carry out their duties properly. Past studies prove the influence of supervision on teacher performance ( Prasetyono et al., 2018), and organizational culture also affects teacher performance ( Emengini et al., 2020). Therefore, we propose hypothetic research model as Figure 1.

**Hypotheses**

1: Academic supervision has a significant positive effect on teacher commitment.
2: Academic supervision has a significant positive effect on teacher performance.
3: Managerial competence has a significant positive effect on teacher commitment.
4: Managerial competence has a significant positive effect on teacher performance.
5: Organizational commitment has a significant positive effect on teacher performance.
6: Teacher empowerment has a significant positive effect on teacher commitment.
7: Teacher commitment has a significant positive effect on teacher performance.
8: Academic supervision has a significant positive effect on teacher performance mediated by teacher commitment.
9: Managerial competence has a significant positive effect on teacher performance mediated by teacher commitment.
10: Teacher empowerment has a significant positive effect on teacher performance mediated by teacher commitment.

## Methods

The present research uses quantitative methods with surveys. The research variables include five variables: the managerial competence of the principal, teacher empowerment, academic supervision, teacher performance, and commitment.

The respondent population of this study includes teachers under the auspices of the Ministry of Religious Affairs who were active and registered on the Sistem Informasi Pendidik dan Tenaga Kependidikan (SIMPATIKA) website in semester 1 of the 2020/2021 academic year in West Kalimantan and East Java. A cluster random sampling technique was used based on the large number of respondents involved in this study, respondents came from teacher on East Java (38 districts and West Kalimantan province (14 districts). The research period lasted for seven months running from February 2022 to August 2022.

Research instruments were adopted and modified from regulations and relevant research results. Data were collected using an online questionnaire including five Likert scale options: (1) strongly disagree, (2) agree, (3) cannot decide, (4) agree, and (5) strongly agree. Validity and reliability of the measurement depend on the score obtained in one group sample, changes in the other group will change the quantitative value of validity and reliability. Validity and reliability was performed directly on one group of participants, with a large sample size - invalid and unreliable items were excluded from the analysis and, re-analysis was conducted only on valid and reliable items ( Hadi, 2004), so the present research using indicator validity, not instrument validity, invalid and unreliable indicators are detailed reported and deleted and then re-analyzed only for valid indicators. This research instrument was adapted from another instrument for several reason; 1) indicators are known or pre-tested (Regulation or research findings, 2) large sample with different district and culture, 3) PLS SEM is different from covariance-based SEM which uses theory to be confirmed, PLS SEM (smartPLS) can be used to confirm existing theories or answer hypotheses.

The questionnaire can be found in the Extended data. There are five constructs of this research: managerial competence, academic supervision, teacher empowerment, teacher performance and teacher commitment. The managerial competence and academic supervision indicators were adapted from the Decree of the Ministry of Educational and Culture of Republic Indonesia number 13 Year 2007 concern about Standards for School/Madrasah Principals ( Republik, 2007). There were 16 indicators (pp.5) for managerial competence, while for supervise competence, there were 3 indicators (pp.7). The present study uses 6 indicators of managerial competence (MC1 to MC6) and 6 indicators for academic supervision (AS1 to AS6). The teacher empowerment indicators were modified and adapted from Short’s teacher empowerment scale ( Short, 1992), consisting of involvement in decision-making, impact, status, autonomy, professional development opportunities, and self-efficiency. There were 11 indicators (TE1 to TE11) used for measures. The teacher performance indicators were adapted from APKG (Alat Penilai Kinerja Guru/Teacher performance assessment tool) sering digunakan seperti ( Aryana et al., 2022; Dahlia & Afriadi, 2020), and this indicator assesses teachers starting from preparation/planning, implementation, and evaluation. Teacher performance indicators of the present study uses 6 indicators (TP1 to TP6). Several indicators in APKG are merged for effectiveness and efficiency, and teachers are light when completing questionnaires. The teacher commitment (TC) instrument was modified from Lei Mee Thien, Nordin Abd Razak, and T. Ramayah instruments ( Thien et al., 2014). They made 13 indicators of teacher commitment used for teachers in Malaysia. The dimensions measured were commitment to students, teaching, school, and profession. The present study used 7 indicators for teachers (TC1-TC7).

Model measurements are performed by calculating the validity and reliability of the instrument. The indicator was assessed with three measurements: 1) indicator loading and internal consistency reliability, 2) convergent validity, and 3) discriminant validity (Hair et al., 2019). Internal consistency reliability was assessed with Cronbach Alpha, Composite Reliability (CR), and Average of Extracted Variants (AVE). While structural model measurement of the present study was performed by assessing the collinearity, coefficient of determination (R2), effect size (F2), predictive relevance (Q2) using the blindfolding procedure, and path coefficient.

*First*, A collinearity was used to test whether this model was worth using. If an instrument’s Varian Inflation Factor (VIF) value is less than 3 for the inner model and less than 10 for the outer model, it can move on to the next step ( Sarstedt & Cheah, 2019). *Second*, The coefficient of determination (R2). The R2 value can be used to determine the goodness of fit (GoF) test. A variation in the proportions of endogenous factors that external variables can predict is the coefficient of determination from 0 to 1. Values range from significant (0.75) to moderate (0.50) to weak (0.25) ( Chin, 1998). *Third*, changes in coefficient of determination (R2) values are used to determine effect size (F2). In order to determine if exogenous latent variables have a significant impact on endogenous variables, this change in value is used ( Ghozali, 2014). Cohen (1998) also divided into a minimal impact (0.02); a moderate impact (0.15); and a considerable impact (0.35); on the structural level ( Chin, 1998). *Fourth* is predictive relevance. The Stone-Geisser (Q2) test is a statistical test to measure how well the model and the resulting parameters produce the observation value (predictive relevance). If the Q2 value is greater than 0, then the model has predictive relevance. *Fifth* is the path coefficient. The calculation of the path coefficient between endogenous and exogenous constructs was performed with 5000 bootstrapping.

### Ethical considerations

The institute for research and community services of Pontianak State Institute for Islamic Studies as ethical committee has approved the study (ethics committee approval number: B-415.1/In.15/LP2M/HM.01/11/2022). Researchers visited on a pre-informed date and obtained permission from Head Office of Ministry of Religious Affairs (MORA) district after explaining the purpose of this study. Teachers who participated in this study were provided with a consent form when opening the questionnaire page. The research instrument was approved by Institutional Review Board (approval number B-43.1/In.15/LP2M/HM.01/2/2022). Teachers who filled out the questionnaire thus agreed to participation in the study, and those who did not participate did not agree (the consent form is provided as *extended data*).

### Model measurement

The measurement model employed in this study uses three measurements: 1) an item validity measurement using indicator loading and internal consistency measurement using Cronbach’s alpha, composite reliability and the average variance extracted (AVE); 2) a convergent validity measurement; and 3) a discriminant validity measurement. First, the validity of an indicator is determined from the value of the loading factor based on SmartPLS output; this value shows the correlation between an indicator and its construct. An indicator with a low value indicates that it does not work in the measurement model, while a high value is said to be valid.

Second, internal consistency measurement or construct reliability is used to measure the internal reliability of variable constructs, and the measurement uses composite reliability (CR) and Cronbach’s alpha. Composite reliability values of between 0.6 and 0.7 are considered to indicate good reliability ( Sarstedt et al., 2021), while Ghozali recommends composite reliability values of above 0.6 ( Ghozali, 2011). In addition, the value of the average variance extracted (AVE) can be used as a guideline for the reliability of a construct. The average variance extracted (AVE) is declared eligible if the value is above 0.50. An AVE value of > 0.50 or higher indicates that a construct can account for more than its indicator variance; conversely, an AVE is less than 0.50 indicates that on average, more variance remains in the item error than the construct describes ( Henseler et al., 2009). More details about the validity and reliability tests can be seen in Table 1. For more validity can be seen also on Fornel-Larcker criterion one Table 2.

**Table 1.** Outer loading and internal reliability.

	Indicators	Loading factors	Cronbach's Alpha	rho_A	Composite reliability	Average Variance Extracted (AVE)	Consideration
Academic Supervision	AS1	0.757	0.903	0.906	0.925	0.674	Valid and realiable
	AS2	0.821					
	AS3	0.813					
	AS4	0.837					
	AS5	0.868					
	AS6	0.825					
Managerial Comptence	MC1	0.806	0.911	0.913	0.931	0.693	Valid and realiable
	MC2	0.837					
	MC3	0.840					
	MC4	0.841					
	MC5	0.846					
	MC6	0.824					
Teacher Commitment	TC1	0.743	0.829	0.831	0.875	0.540	Valid and realiable
	TC2	0.694					
	TC3	0.682					
	TC5	0.809					
	TC6	0.696					
	TC7	0.775					
Teacher Empoweremnt	TE1	0.741	0.845	0.851	0.883	0.519	Valid and realiable
	TE10	0.667					
	TE11	0.771					
	TE3	0.671					
	TE6	0.759					
	TE8	0.652					
	TE9	0.771					
Teacher Performance	TP1	0.765	0.876	0.877	0.906	0.617	Valid and realiable
	TP2	0.805					
	TP3	0.810					
	TP4	0.767					
	TP5	0.801					
	TP6	0.761					

**Table 2.** Fornel-Larcker criterion.

	Academic Supervision	Manajerial Competence	Teacher Commitment	Teacher Empowerment	Teacher Performance
Academic Supervision	**0.821**				
Manajerial Competence	0.568	**0.833**			
Teacher Commitment	0.431	0.502	**0.735**		
Teacher Empowerment	0.480	0.529	0.634	**0.720**	
Teacher Performance	0.488	0.547	0.592	0.722	**0.785**

Third, discriminant validity testing involves comparing the AVE to the root of the AVE or Fornell-Larcker criterion. From the previous table, the AVE value of academic supervision is 0.674, that of managerial competence is 0.693, that of teacher commitment is 0.540, that of teacher empowerment is 0.519, and that of teacher performance is 0.617. When compared to the AVE root in the following Fornell-Larcker table, the AVE root is greater than its correlation with the construct, which means it has discriminant validity.

Discriminant validity can also be determined from the cross-loading value of each construct. If the loading value of an item/indicator is greater than the cross-loading value, it is said to have discriminant validity. In addition to these criteria, Ghozali added that the *cross-loading* value should be greater than 0.7.

**Table 3.** Cross-loading.

	Academic Supervision	Manajerial Competence	Teacher Commitment	Teacher Empowerment	Teacher Performance
**AS1**	0.757	0.420	0.339	0.372	0.356
**AS2**	0.821	0.491	0.357	0.379	0.411
**AS3**	0.813	0.396	0.306	0.370	0.351
**AS4**	0.837	0.505	0.393	0.414	0.420
**AS5**	0.868	0.478	0.361	0.421	0.410
**AS6**	0.825	0.489	0.358	0.405	0.445
**MC1**	0.455	0.806	0.381	0.412	0.420
**MC2**	0.449	0.837	0.416	0.414	0.417
**MC3**	0.488	0.840	0.448	0.463	0.463
**MC4**	0.449	0.841	0.443	0.442	0.455
**MC5**	0.489	0.846	0.404	0.462	0.471
**MC6**	0.503	0.824	0.413	0.447	0.501
**TC1**	0.323	0.372	0.743	0.500	0.444
**TC2**	0.364	0.391	0.694	0.503	0.434
**TC3**	0.295	0.353	0.682	0.449	0.413
**TC5**	0.334	0.398	0.809	0.499	0.467
**TC6**	0.264	0.311	0.696	0.386	0.398
**TC7**	0.305	0.375	0.775	0.436	0.445
**TE1**	0.354	0.452	0.479	0.741	0.550
**TE10**	0.330	0.292	0.412	0.667	0.405
**TE11**	0.352	0.401	0.498	0.771	0.622
**TE3**	0.373	0.433	0.462	0.671	0.477
**TE6**	0.352	0.399	0.470	0.759	0.592
**TE8**	0.312	0.335	0.397	0.652	0.450
**TE9**	0.350	0.341	0.468	0.771	0.507
**TP1**	0.391	0.447	0.455	0.544	0.765
**TP2**	0.381	0.432	0.486	0.586	0.805
**TP3**	0.405	0.448	0.477	0.602	0.810
**TP4**	0.345	0.398	0.469	0.572	0.767
**TP5**	0.401	0.438	0.492	0.582	0.801
**TP6**	0.376	0.414	0.406	0.510	0.761

For example, the loading value of AS1 is 0.757, which is greater than the value of AS1 for managerial competence (0.420), teacher commitment (0.339), teacher empowerment (0.372) and teacher performance (0.356). In the table above, all loading values are greater than the cross-loading values, which means that they have good discriminant validity. To strengthen discriminant validity further, we also use the Heterotrait-Monotrait (HTMT) criterion. HTMT values should be less than 0.9 to ensure reflective construct validity ( Henseler et al., 2009). All values of the HTMT construct for this study are below 0.9, meaning that discriminant validity requirements are met. The detail HTMT table as shown on Table 4.

**Table 4.** HTMT.

	Academic Supervision	Manajerial Competence	Teacher Commitment	Teacher Empowerment	Teacher Performance
**Academic Supervision**					
**Manajerial Competence**	0.622				
**Teacher Commitment**	0.493	0.574			
**Teacher Empowerment**	0.550	0.599	0.751		
**Teacher Performance**	0.546	0.611	0.693	0.830	

### Structural model measurements

**Figure 2.**
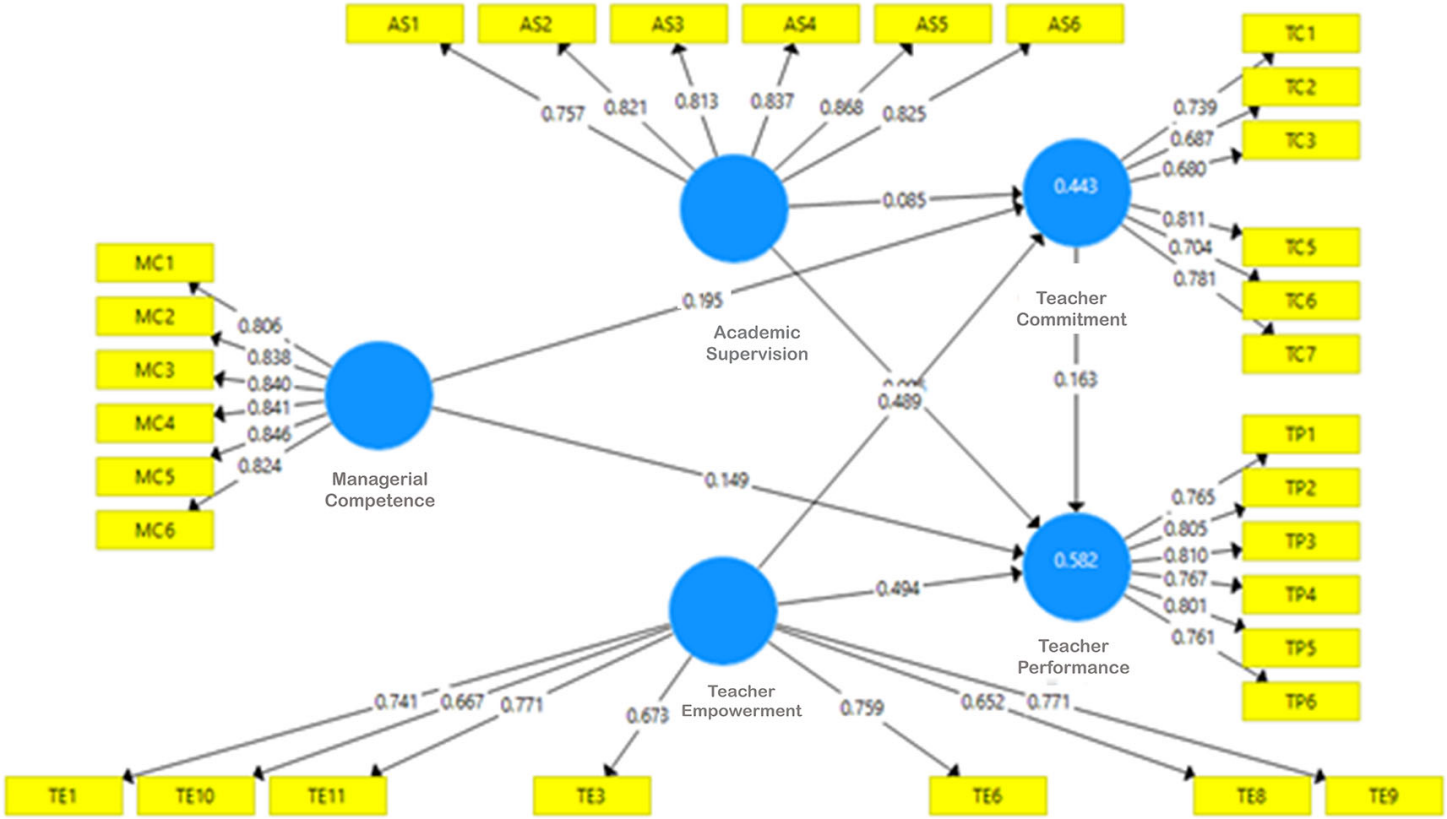
Final model.

A structural model serves to predict the relationships between constructs used in a study. We apply the model using bootstrapping and blindfolding procedures using SmartPLS. The procedure is used to determine the coefficient of the determinant (R2)/ *R-square* of the exogenous construct, effect size, and predictive relevance/Q2 of the model. One of the conditions for testing using PLS is a lack of multicollinearity; therefore, before other tests are carried out, whether an intercorrelation between latent/exogenous variables is analyzed. Final model after validity, reliability and collinearity test show on Figure 2 as follows.

### Collinearity test

Collinearity refers the existence of a close relationship between independent/exogenous variables, as a strong relationship can interfere with the precision of predictions of the proposed model. Therefore, a good model must be free of collinearity. The presence or absence of collinearity can be determined from the tolerance value and variant inflation factor (VIF). The VIF value must be below 5; if it is above 5, multicollinearity exists ( Sarstedt et al., 2021). Collinearity checking is performed to calculate the inflation factor (VIF) variant provided by SmartPLS.

The VIF table shows no multicollinearity between the variables of this study. From Table 5, there is no multicollinearity between the variables of this study.

**Table 5.** VIF.

	Teacher Commitment	Teacher Performance
Academic Supervision	1.580	1.580
Manajerial Competence	1.689	1.689
Teacher Empowerment	1.488	1.488

### Path coefficient

We next determine the potential direct or indirect influence of exogenous variables on endogenous variables. SmartPLS provides tables for both direct and indirect path analysis. From the output of the path coefficient table, it can be concluded that academic supervision has a significant positive direct effect on teacher commitment and teacher performance, managerial competence has a significant positive direct effect on teacher commitment and performance, and teacher empowerment has a significant positive direct effect on teacher commitment and performance. Table 6 shows these results in detail.

**Table 6.** Path coefficient

	*β*	Sample Mean	Standard Deviation	T Statistics	*P-value* s	Consideration
Academic Supervision -> Teacher Commitment	0.085	0.085	0.022	3.792	0.000	Positive-significant
Academic Supervision -> Teacher Performance	0.096	0.097	0.022	4.416	0.000	Positive-significant
Manajerial Competence -> Teacher Commitment	0.195	0.195	0.024	8.046	0.000	Positive-significant
Manajerial Competence -> Teacher Performance	0.149	0.149	0.023	6.561	0.000	Positive-significant
Teacher Commitment -> Teacher Performance	0.163	0.163	0.023	7.263	0.000	Positive-significant
Teacher Empowerment -> Teacher Commitment	0.489	0.488	0.022	22.601	0.000	Positive-significant
Teacher Empowerment -> Teacher Performance	0.494	0.494	0.022	22.301	0.000	Positive-significant

Regarding the indirect influence of the three variables with the mediation of teacher commitment, Table 7 shows the beta coefficient, *t-statistics* and *p-values.* The SmartPLS output shows that all variables have a significant positive effect on teacher performance with the mediation of teacher commitment.

**Table 7.** Indirect effect (mediating role).

	*β*	Sample Mean (M)	Standard Deviation (STDEV)	T Statistics (|O/STDEV|)	*P-value* s	Consideration
Academic Supervision -> Teacher Commitment						
Academic Supervision -> Teacher Performance	0.014	0.014	0.004	3.178	0.001	Positive-significant
Manajerial Competence -> Teacher Commitment						
Manajerial Competence -> Teacher Performance	0.032	0.032	0.006	5.588	0.000	Positive-significant
Teacher Commitment -> Teacher Performance						
Teacher Empowerment -> Teacher Commitment						
Teacher Empowerment -> Teacher Performance	0.080	0.080	0.011	7.050	0.000	Positive-significant

**Coefficient of determination (R ^2^)**

To predict the effect of exogenous variables on endogenous variables in the structural model of this study, we use the coefficient of determination, which is a means to calculate how much an endogenous construct can be explained by an exogenous construct. The R-squared value ranges from 0 to 1. *R-square* adjusted, the *R-square* adapted to the standard error, is more precise because it involves error calculation. Table 8 shows the output of the calculation results of the coefficient of determination. An *R-square* value of 0.67 is considered high, a value of 0.33 is moderate and a value of 0.19 is low ( Chin, 1998). Table 8 shows the *R-square* results of this study.

**Table 8.** R-Square.

	R Square	R Square Adjusted
Teacher Commitment	0.445	0.444
Teacher Performance	0.567	0.566

To increase confidence in the *R-square* value, the influence of exogenous variables on individual variables, including substantive variables, should be considered ( Ghozali, 2014). This study also used *F-square* to calculate the effect size. The value of f2 is used to determine the effect of exogenous variables on endogenous variables, including strong, moderate and weak effects. If the *F-square* value is less than 0.15 (0.02 ≤ f2 < 0.15), the effect is weak; a value between 0.16 and 0.35 (0.15 ≤ f2 < 0.35) denotes a moderate effect; and a value greater than 0.35 (0.35 ≤ f2) denotes a strong effect. Table 9 shows the *F-square* output in detail.

**Table 9.** F-square.

	Teacher Commitment	Teacher Performance
Academic Supervision	0.008	0.018
Manajerial Competence	0.040	0.045
Teacher Empowerment	0.291	0.510

To test predictive relevance, this study uses the Stone-Geisser test (Q2); this calculation is used to see how well a model can predict certain parameters. Results below 0 denote a model with no predictive relevance. In PLS-SEM, predictive relevance is tested using blindfolding, which is an analysis of data used to determine the relevance of predictions produced by a construct; the process uses the *Q-square* value as a reference. Ghozali created three blindfolding levels: less (0.02), moderately (0.15) and highly predictive (0.35) ( Ghozali, 2014). The complete blindfolding calculation results are given in Table 10.

**Table 10.** Predictive relevance (Q2).

	SSO	SSE	Q ^2^(=1-SSE/SSO)
Teacher Commitment	13218.000	10116.566	0.235
Teacher Performance	13218.000	8516.300	0.356

## Discussion

This study aims to determine the influence of academic supervision, managerial competence, and teacher empowerment on teacher commitment directly and with the mediation of teacher performance. Calculations made via bootstrapping with 5,000 repetitions reveal an influence of academic supervision on teacher commitment directly and with the mediation of teacher performance. Principal managerial competence also directly affects teachers’ commitment and the mediation of teacher performance. A teacher’s performance also affects a teacher’s commitment.

We test how much influence exogenous constructs have on endogenous constructs by calculating the coefficient of determination (R-square). Overall, the influence of the exogenous construct shows an *R-square* of 0.445 with an adjusted *R-square* of 0.444. The figure shows that 44.4% of teacher commitment can be explained by academic supervision, managerial competence, and teacher empowerment. The three constructs also affect teacher performance, with an *R-square of* 0.567 and an *R-square* of 0.566. To increase the degree of trust, we also used an F test ( *F-square*). Academic supervision’s effect on teacher commitment is 0.008 and the effect on teacher performance is 0.018. The effect of management competence on teacher commitment is 0.040, and the effect on teacher performance is 0.045.

Meanwhile, the effect of teacher empowerment on teacher commitments is valued at 0.291, and the effect on teacher performance is valued at 0.510. To determine how well the model predicts the research constructs, a blindfolding stone-Gaisser (Q2) calculation is used. SmartPLS output shows a figure of 0.235 for teacher commitment, while for teacher performance, we obtain a figure of 0.356. This output shows that predictive relevance is in the moderate category (<0.15).

Hypothesis 1 posits a significant positive effect of academic supervision on teacher commitment. This hypothesis is accepted we found a *β* value of 0.085, *t-statistic* of 3,792, and *p-value* of 0.000. A *t-statistic*al value greater than 1.96 indicates a positive influence of academic supervision on teachers’ organizational commitment that is significant with a *p-value* below 0.05. This finding is consistent with Irawan *et al.* (2018) who report that academic supervision affects teachers’ organizational commitment. Academic supervision indicators of teacher commitment include assistance from principals and supervisors in syllabus/teaching preparation (AS1), creating teaching materials, media and tests (AS2), material delivery (AS3), learning strategies and media (AS4), the evaluation of learning processes and outcomes (AS5), and question item analysis (AS6).

Study conducted by Berjoaui and Karami posits that distributed leadership serves as a conduit to enhancing teacher commitment, whereas Rachmawati and Suyatno focused their research on the role of the principal’s competence in fostering job satisfaction and teacher commitment. This present study unequivocally asserts that the academic supervision executed by school principals—utilizing the six delineated indicators—directly impacts teacher performance.

Regarding Hypothesis 2, academic supervision is found to have a direct influence on teacher performance, as the SmartPLS output shows a *β* value of 0.096, *t-statistic* of 4.416 and *p-value* of 0.000. A *t-statistic* above 1.96 indicates that a construct has an influence, and *p-value* below 0.05 indicate a significant effect. The findings of this study are consistent with the results of a study conducted in Malaysia finding a relationship between supervision and teacher performance ( Hoque et al., 2020). The findings also corroborate Hidayatullah and colleagues’ (2020) finding that supervision influences teacher performance. Aprida et al. (2020) also reported that school administration and supervision affect teacher performance. Seven indicators of academic supervision affect the performance of teachers in syllabus/teaching preparation (TP1), material development and media and learning evaluation (TP2), the use of appropriate methods (TP3), the use of learning media (TP4), the evaluation of processes and results (TP5), the analysis of question items and learning evaluation (TP6).

The differentiation of this study with other represented by six distinct indicators, stands in stark contrast to the research conducted by Hoque, Hidayatullah, and Aprida. The present study differentiates itself not only through the use of these indicators, but also in terms of sample size, the parent organizations of the teachers, and the geographical region under investigation. Moreover, our research diverges from Kusumawati’s approach, which employs school action research to enhance teacher commitment. The respondent selection also differentiates this study from previous work; while earlier studies focused on a single school, this study encompasses samples from two provinces in Indonesia on Islamic elementary schools, Islamic junior high schools, and Islamic junior high schools.

Hypothesis 3, which states that a principal’s managerial competence affects a teacher’s organizational commitment, is accepted, as the SmartPLS output shows a *β* value of 0.195, *T-statistic* of 8,046, and *p-value* of 0.000. The findings of this study are consistent with those of Rachmawati and Suyatno, who revealed that a principal’s interpersonal and technical competence significantly affects teachers’ commitment ( Rachmawati & Suyatno, 2021). The difference with this present study is in the variables, indicators and respondents used. However, the dimensions and indicators used are different from this study.

Hypothesis 4 states that a principal’s managerial competence has a direct significant effect on teacher performance, which is accepted due to the following statistical results: a *β* value of 0.149, *T-statistic* of 6.561, and *p-value* of 0.000. A *t-statistic*al score greater than 1.96 indicates a direct influence of a principal’s managerial competence on teacher performance. A *p-value* below 0.05 indicates that the effect is significant. This finding is consistent with Rahman Tanjung *et al.* (2021), who found an influence of a principal’s managerial competence on teacher performance, and with Susanti (2021), who also reports an effect of a principal’s managerial competence and work climate on teacher performance.

Hypothesis 5 states a significant positive influence of direct teacher commitment on teacher performance, which is accepted from the following statistical results: a *β* value of 0.163, *t-statistic* of 7.263, and *p-value* of 0.000. A *t-statistic*al score greater than 1.96 indicates a direct influence of teacher commitment on teacher performance. A *p-value* below 0.05 indicates a significant effect. This finding is consistent with Agung’s study, which identifies the influence of organizational commitment on performance ( Agung, 2017), and it corroborates The study of Butar *et al.* (2020), which finds an effect of organizational commitment on teacher performance both directly and indirectly. The six indicators influenced by teacher commitment in this study include teachers speaking well of their school/madrasah to others (TC1), alignment between a school’s values and a teacher’s personal values (TC2), commitment to students’ success (TC3), enjoyment of teaching (TC5), a desire to teach (TC6), and feeling that becoming a teacher was the right decision (TC7).

Hypothesis 6 posits a significant positive influence of direct teacher empowerment on teachers’ organizational commitment, which is accepted due to the following statistical results: a *β* value of 0.489, *t-statistic* of 22.601 and *p-value* of 0.000. A *t-statistical* score greater than 1.96 indicates a direct influence of teacher commitment on teacher performance. A *p-value* below 0.05 indicates a significant influence. These findings corroborate the work of Lee and Nie (2014) and Toremen *et al.* (2011), who found an effect of teacher empowerment on teacher commitment. The professional dimension of growth, which refers to the ability of teachers to operate in a professional school environment (TE1) and cooperate and collaborate with other teachers both in and outside of school (TE3), contributes to teacher commitment, as does the status dimension of teacher empowerment, which includes obtaining in-depth knowledge and understanding of the field of science (TE6). One indicator of the autonomy dimension is teachers’ freedom to select strategies, teaching materials and their own materials (TE8). Impact dimensions including the ability to influence other teachers and students (TE9), teachers’ confidence in their impact on their school/madrasah (TE10), and the ability to complete schoolwork well (TE11) also increase teacher commitment.

Hypothesis 7 posits a significant positive influence of direct teacher empowerment on teacher performance, which is accepted due to the following statistical results: a *β* value of 0.494, *t-statistic* of 22.301 and *p-value* of 0.000. A *t-statistic*al score greater than 1.96 indicates a direct influence of teacher commitment on teacher performance. A *p-value* below 0.05 indicates a significant influence. The seven teacher dimensions (TE1, TE3, TE6, TE8, TE9, TE10 and TE11) have a significant positive effect on teachers’ performance in compiling a teaching syllabus/preparing for lessons (TP1), material development and media and evaluation (TP2), the use of appropriate methods for learning materials (TP3), the use of learning media that are appropriate to the material and connect with the real world (TP4), evaluation of learning processes and outcomes (TP5) and the implementation of question analysis items and evaluation. These findings are consistent with studies conducted by Lutsilili and East Constituency, which reveal effects of teachers’ empowerment on their performance ( Constituency et al., 2014; Lutsilili et al., 2012).

Hypothesis 8 posits a significant positive influence of academic supervision on teacher performance through the mediation of teacher commitment, which is accepted due to the following statistical results: a *β* value of 0.014, *t-statistic* of 3.178 and *p-value* of 0.001. A *t-statistic* score greater than 1.96 indicates a direct influence of teacher commitment on teacher performance. An accepted *p-value* below 0.05 indicates a significant influence. This finding is consistent with the results of Susana, who finds effects of supervision and teacher commitment on teacher performance ( Susana, 2018). The commitment variable used in this study is mediation, while Susana studied exogenous variables, which means that teacher commitment affects teachers either as a mediation variable or directly.

Hypothesis 9 posits a significant positive influence of principal managerial competence on teacher performance through the mediation of teacher commitment, which is accepted due to the following statistical results: a *β* value of 0.032, *t-statistic* of 5.588 and *p-value* of 0.000. A *t-statistic* score greater than 1.96 indicates a direct influence of teacher commitment on teacher performance. A *p-value* below 0.05 indicates a significant influence. These findings are consistent with previous studies showing that the managerial competence of school principals consistently affects achievement and other aspects ( Grissom & Loeb, 2011). These findings also corroborate the results of Paturusi, who found an effect ( Paturusi, 2017), reinforcing the relationship between managerial competence and teacher performance.

A previous study by Fajirin Husnu Haitami, Achmad Suriansah, and Suhaimi employed teacher commitment as a mediating variable for the influence of school-based management on teacher performance. In contrast, our research introduces a novel approach by demonstrating the impact of managerial competence on teacher performance, with teacher commitment serving as the mediating variable.

Hypothesis 10 states that teacher empowerment significantly influences teacher performance through the mediation of teacher commitment, which is accepted due to the following statistical results: a *β* value of 0.080, *t-statistic* of 7,050, and *p-value* of 0.000. A *t-statistic*al score greater than 1.96 indicates a direct influence of teacher commitment on teacher performance. A *p-value* below 0.05 indicates a significant influence. The seven teacher empowerment indicators used in this study positively and significantly affected teacher performance by intervening teacher commitment. Being in a professional community of fellow teachers to learn influences teacher commitment ( Lee et al., 2011) equally. This indicator is also a teacher empowerment dimension. Empowered teachers tend to speak well of their school/madrasah to others (TC1). They also feel alignment between the values adopted by their school and their personal values (TC2), have a mission to make their students successful (TC3), enjoy teaching (TC5), always wanted to be a teacher (TC6) and feel that becoming a teacher was the right decision (TC7).

Bogler and Somech primarily focused on the impact of teacher empowerment on teachers’ organizational commitment. In contrast, our study concentrates on the teacher’s commitment to teaching. As a result, all commitment indicators employed in our study derive from the teacher’s teaching commitment. Moreover, this study substantiates Limon’s findings, asserting that teacher commitment can positively mediate the effect of teacher empowerment on teacher performance. Limon’s research utilized commitment indicators adapted from Meyer’s model in Turkey, which measured affective, continuance, and normative commitments. In contrast, our research applies the concept of teacher commitment in the realm of teaching.

## Conclusion

Teacher commitment can be increased through academic supervision from the principal and supervisor, managerial competence from the principal, and teacher empowerment. These three factors directly and indirectly affect teachers’ commitment. Independent teacher performance can predict teacher commitment. In addition, teacher performance moderates the impacts of academic supervision, managerial competence, and teacher empowerment on teacher commitment. Among the variables used in the present study, teacher empowerment is the most significant variable influencing teacher commitment and performance, while supervision has the weakest influence. Therefore, school principals, supervisors, and the government are advised to improve programs that empower teachers because they influence teacher commitment and performance. Academic supervision, managerial competence, and teacher empowerment are the three factors directly and indirectly affect teachers’ commitment, and teacher performance can predict teacher commitment.

In addition, teacher performance moderates the impacts of academic supervision, managerial competence, and teacher empowerment on teacher commitment. Among the variables used in this study, teacher empowerment affects teacher commitment and performance most, while supervision has the weakest effect. Therefore, principals, supervisors, and governments are advised to improve programs that empower teachers because they affect teacher commitment and performance.

Theoretically, the findings of this study offer significant implications for our understanding of teacher commitment as a mediating variable influencing teacher performance, particularly in the context of teachers affiliated with the Ministry of Religious Affairs of the Republic of Indonesia. The findings of this study demonstrate that teachers’ commitment to their duties and responsibilities exerts a significant influence on their performance. Additionally, teacher commitment serves as a mediator for other variables that could potentially be exploited to improve teacher performance. These findings illuminate the multifaceted role of teacher commitment, acting not only as a direct contributor to performance but also as a mediator for other influences on teacher performance.

This study suggests that school principals should prioritize enhancing both teacher commitment and performance. In the present research, we have incorporated academic supervision, managerial competence, and teacher empowerment as variables. Future researchers might consider exploring other variables that could influence teacher performance, with teacher commitment serving as the mediating factor.

### Limitations

The present study is limited by its sample, which, although quite large, cannot be generalized to the rest of Indonesia. However, theoretically, this study proves the effects of academic supervision, managerial competence and teacher empowerment on teacher performance both directly and through the mediation of teacher commitment.

### Suggestions

Further work can determine other factors hypothesized to affect teacher performance and commitment, since the variables used in this study represented 44.4% of the identified effects, while 45.6% of the effects were attributed to other variables.

## Data Availability

Harvard Dataverse: “Effect of Supervision, Managerial Competence, Empowerment on Performance; Commitment as intervening effect”, https://doi.org/10.7910/DVN/CMLWOU ( Muttaqin, 2022). This project contains the following underlying data:
- Data CSV for SmartPLS.csv -
Valid instrument This project contains the following extended data:
- Questionnaire in English and Indonesia.pdf -
Research Contract.pdf
-
Ethical Commission Approval.pdf
-
Letter from the Ministry of Religious Affairs Blitar disctric.pdf
-
Letter from Head of The Office of Religious Affairs of Bengkayang.pdf
-
Letter from Section Chief of Madrasah’s Education of The Office of Religious Affairs of Surabaya City number B-5022/Kk.13.29.2/PP.00/07/2022 -
Penma Blitar request permission
-
Letter from the Ministry of Religious Affairs Blitar disctric.pdf
- Validity and reliability test.xlsx - Bootstrap 5000.xlsx - Blindfolding.xlsx Data are available under the terms of the Creative Commons Zero “No rights reserved” data waiver (CC0 1.0 Public domain dedication).
