# Supplementary material for: The effect of academic supervision, managerial competence, and teacher empowerment on teacher performance: the mediating role of teacher commitment
Source: F1000Res. 2023 Sep 19;12:743. Originally published 2023 Jun 26. [Version 2] doi: 10.12688/f1000research.128502.2 (PMC10517307; doi:10.12688/f1000research.128502.2)
Supplement: Peer review history

# Reviewer report: Reviewer response for version 2

**DOI:** 10.5256/f1000research.153809.r207688

**Published:** 2023-09-22

**Authors:** MD. Rahat Khan

**Affiliations:** 1 Department of Business Administration, City University, Dhaka, Bangladesh

I am happy with the current version of this manuscript.

Is the work clearly and accurately presented and does it cite the current literature?

Yes

If applicable, is the statistical analysis and its interpretation appropriate?

Yes

Are all the source data underlying the results available to ensure full reproducibility?

Yes

Is the study design appropriate and is the work technically sound?

Partly

Are the conclusions drawn adequately supported by the results?

No

Are sufficient details of methods and analysis provided to allow replication by others?

Partly

Reviewer Expertise:

Quantitative and Qualitative Research; Business and Management; Strategic issues in business.

I confirm that I have read this submission and believe that I have an appropriate level of expertise to confirm that it is of an acceptable scientific standard.

# Reviewer report: Reviewer response for version 1

**DOI:** 10.5256/f1000research.141097.r181578

**Published:** 2023-07-20

**Authors:** Idi Warsah, Muhammad Uyun

**Affiliations:** 1 Institut Agama Islam Negeri Curup, Curup, Bengkulu, Indonesia; 2 Educational Psychology, Universitas Islam Negeri Raden Fatah Palembang, Palembang, South Sumatra, Indonesia

This paper, as a whole, has been well-written and the theoretical and data-based details have been appropriate. However, when addressing that the instruments used had been told to be valid, the researchers need to prove it by giving the scores of validities and reliability of each instrument within the paper (though the detailed validity computation has been enclosed).

 Also, the researchers need to discuss the results by providing argumentations comparing the present results and the previous ones in terms of differences. Most of comparison made in the discussion showcases that the present results are consistent or support the previous ones. If so, what is something new to be highlighted from this study. The researchers should make sure that there are parts of the present research different from the preceding ones to cast light some novel parts of this study.

 Lastly, in the section of recommendation, the authors emphasized that there were some external factors affecting the results, so the authors recommend others to conduct future's studies working with such factors. Please give some possible examples of such factors so that further researchers could find their ways to go.

Is the work clearly and accurately presented and does it cite the current literature?

Yes

If applicable, is the statistical analysis and its interpretation appropriate?

Yes

Are all the source data underlying the results available to ensure full reproducibility?

Yes

Is the study design appropriate and is the work technically sound?

Yes

Are the conclusions drawn adequately supported by the results?

Yes

Are sufficient details of methods and analysis provided to allow replication by others?

Yes

Reviewer Expertise:

Education, Educational Psychology, Multiculturalism

We confirm that we have read this submission and believe that we have an appropriate level of expertise to confirm that it is of an acceptable scientific standard.

# Reviewer report: Reviewer response for version 1

**DOI:** 10.5256/f1000research.141097.r181580

**Published:** 2023-07-14

**Authors:** MD. Rahat Khan

**Affiliations:** 1 Department of Business Administration, City University, Dhaka, Bangladesh

Dear Author

 Thank you so much for giving me the chance to read your paper. I found the paper interesting, but there need some adjustments that may enrich the quality and readability of this manuscript.

1. The problem statement is incorrectly designed in the 'Introduction.' You need to develop research questions based on your identified problem. The problem statement is not in-depth; you need to explain the statement based on global context and then focus on various developing country perspectives. Go and search the different databases to strengthen your problem statement.
2. The section “literature review” is missing. Develop the section first to deep dive into your problem statement (add the latest citations to the research context) in detail and then develop the "Theoretical framework and hypotheses". Please read and cite the following papers to learn how to differentiate the “literature review” and “Theoretical framework and hypotheses” and learn the recent trend of writing hypothesis development.
  Khan, M. R., Rana, S., & Hosen, M. I. (2022). Impact of trustworthiness on the usage of m-banking apps: A study on Bangladeshi consumers. Business Perspectives and Research, 10(2), 234-250.
  Khan, M. R., & Roy, S. K. (2023). Do primary HR functions model work in emerging economies? Sustainable compact perspective for Bangladeshi RMG industry. *Review of International Business and Strategy*, *33*(2), 328-341.
3. More information is required in the 'Methodology" section. There are some mismatches in the 'Methodology" and "Result Analysis" sections. Please see the suggested paper to develop the section. The mediation effect analysis is confusing and the tabulation of the entire hypothesis outcomes can be presented in the same table. See the following papers for mediation analysis writing.
  Mitra, A., & Sur, S. (2023). Subjective Knowledge, Perceived Ability, Motivation, and Age: A Mediated Moderation Model of Antecedents of Stock Market Investors’ Information Search. *Global Business Review*, 09721509221146062.
  Al Ahad, A., & Khan, M. R. (2020). Exploring the mediating effect of demographic attributes on the relationship between employee engagement and organizational citizenship behavior. *International Journal of Management and Sustainability*, *9*(1), 11-23.
  “Ethical considerations” is not an essential highlighting heading in a research paper but rather a dissertation writing. Hence this part can be synthesized under the methodology section.
4. The 'Discussion' section is missing and needs to focus more on the novelty of this research. You need to compare the earlier related research to vivid why your research is unique in the international banking business literature.
5. Your conclusion section needs to add more subheadings on the 'Implications' and 'Future Direction of the research' Implication sections should be separated from this section and be fragmented as managerial and theoretical implications of this research.
6. I recommend seeing the suggested articles to develop the overall structure of your paper.

Best wishes

Is the work clearly and accurately presented and does it cite the current literature?

Yes

If applicable, is the statistical analysis and its interpretation appropriate?

Yes

Are all the source data underlying the results available to ensure full reproducibility?

Yes

Is the study design appropriate and is the work technically sound?

Partly

Are the conclusions drawn adequately supported by the results?

No

Are sufficient details of methods and analysis provided to allow replication by others?

Partly

Reviewer Expertise:

Quantitative and Qualitative Research; Business and Management; Strategic issues in business.

I confirm that I have read this submission and believe that I have an appropriate level of expertise to confirm that it is of an acceptable scientific standard, however I have significant reservations, as outlined above.

## References

[1] : Impact of Trustworthiness on the Usage of M-banking Apps: A Study on Bangladeshi Consumers. *Business Perspectives and Research* .2022;10(2) : 10.1177/22785337211001969 234-250 10.1177/22785337211001969

[2] : Do primary HR functions model work in emerging economies? Sustainable compact perspective for Bangladeshi RMG industry. *Review of International Business and Strategy* .2023;33(2) : 10.1108/RIBS-04-2021-0065 328-341 10.1108/RIBS-04-2021-0065

[3] : Subjective Knowledge, Perceived Ability, Motivation, and Age: A Mediated Moderation Model of Antecedents of Stock Market Investors’ Information Search. *Global Business Review* .2023; 10.1177/09721509221146062 10.1177/09721509221146062

[4] : International Journal of Management and Sustainability, Conscientia Beam.2020;9(1) :11-23
